# CIDO ontology updates and secondary analysis of host responses to COVID-19 infection based on ImmPort reports and literature

**DOI:** 10.1186/s13326-021-00250-4

**Published:** 2021-08-28

**Authors:** Anthony Huffman, Anna Maria Masci, Jie Zheng, Nasim Sanati, Timothy Brunson, Guanming Wu, Yongqun He

**Affiliations:** 1 Department of Computational Medicine and Biology, University of Michigan, Ann Arbor, MI 48109 USA; 2 Department of Biostatistics and Bioinformatics, Duke University School of Medicine, Durham, NC 27710 USA; 3 Office of Data Science, National Institute of Environmental Health Sciences, 530 Davis Drive, Research Triangle Park, NC 27560 USA; 4 Department of Genetics, University of Pennsylvania Perelman School of Medicine, Philadelphia, PA 19104 USA; 5 Department of Medical Informatics and Clinical Epidemiology, Oregon Health and Science University, Portland, OR 97239 USA; 6 Unit for Laboratory Animal Medicine, University of Michigan Medical School, Ann Arbor, MI 48109 USA; 7 Department of Microbiology and Immunology, University of Michigan Medical School, Ann Arbor, MI 48109 USA; 8 Center for Computational Medicine and Biology, University of Michigan, Ann Arbor, MI 48109 USA

**Keywords:** ImmPort, COVID-19, SARS-CoV-2, Host-coronavirus interaction, Ontology, Coronavirus infectious disease ontology, CIDO, Metadata, Sex difference, OBO foundry

## Abstract

**Background:**

With COVID-19 still in its pandemic stage, extensive research has generated increasing amounts of data and knowledge. As many studies are published within a short span of time, we often lose an integrative and comprehensive picture of host-coronavirus interaction (HCI) mechanisms. As of early April 2021, the ImmPort database has stored 7 studies (with 6 having details) that cover topics including molecular immune signatures, epitopes, and sex differences in terms of mortality in COVID-19 patients. The Coronavirus Infectious Disease Ontology (CIDO) represents basic HCI information. We hypothesize that the CIDO can be used as the platform to represent newly recorded information from ImmPort leading the reinforcement of CIDO.

**Methods:**

The CIDO was used as the semantic platform for logically modeling and representing newly identified knowledge reported in the 6 ImmPort studies. A recursive eXtensible Ontology Development (XOD) strategy was established to support the CIDO representation and enhancement. Secondary data analysis was also performed to analyze different aspects of the HCI from these ImmPort studies and other related literature reports.

**Results:**

The topics covered by the 6 ImmPort papers were identified to overlap with existing CIDO representation. SARS-CoV-2 viral S protein related HCI knowledge was emphasized for CIDO modeling, including its binding with ACE2, mutations causing different variants, and epitope homology by comparison with other coronavirus S proteins. Different types of cytokine signatures were also identified and added to CIDO. Our secondary analysis of two cohort COVID-19 studies with cytokine panel detection found that a total of 11 cytokines were up-regulated in female patients after infection and 8 cytokines in male patients. These sex-specific gene responses were newly modeled and represented in CIDO. A new DL query was generated to demonstrate the benefits of such integrative ontology representation. Furthermore, IL-10 signaling pathway was found to be statistically significant for both male patients and female patients.

**Conclusion:**

Using the recursive XOD strategy, six new ImmPort COVID-19 studies were systematically reviewed, the results were modeled and represented in CIDO, leading to the enhancement of CIDO. The enhanced ontology and further seconary analysis supported more comprehensive understanding of the molecular mechanism of host responses to COVID-19 infection.

## Background

COVID-19 has posed a series of major crises in global public health. With more than 20,000 confirmed cases and almost 1000 deaths in the European Region on March 12, 2020, the WHO declared the pandemic status of the COVID-19 outbreak. As of May 3, 2021, the COVID-19 pandemic had caused over 140 million confirmed cases with over 3 million deaths worldwide, and 31 million confirmed cases with 565 K deaths in the USA [1]. It is critical to systematically study the molecular mechanisms of COVID-19 disease formation and host responses in order to fully understand, prevent, and treat COVID-19.

To better study and understand the disease mechanism, extensive research has been conducted in a relatively short period of time. With tens of thousands of papers published on host-coronavirus interactions (HCIs), a major bottleneck is how to incorporate all the studies into a more comprehensive understanding of the HCI mechanisms. For example, the ImmPort database provides the data related to immune responses stimulated by various agents including infections and vaccines [2]. As of April 22, 2021, ImmPort has included 7 studies on COVID-19, and 6 of these studies have included unique and large data sets.

The Coronavirus Infectious Disease Ontology (CIDO) is a community-based ontology in the domain of coronaviruses [3]. CIDO covers various coronavirus infectious diseases, with a major focus on COVID-19. The areas of CIDO coverage are broad, including various coronaviruses, hosts, host-coronavirus interactions, phenotypes, vaccine, drugs, epidemiology, etc. As a formal biomedical ontology, CIDO is a human- and computer-interpretable representation of the entities and relationships among the entities in the specific coronavirus infectious disease domain. As of the end of March 2021, the CIDO version 1.0.187 includes over 8111 terms and is continuously updating. Like other ontologies, CIDO allows semantical reasoning and enables humans and machines to make mutually understandable logical inferences. With more studies conducted, it is required to continuously update CIDO. Although manual ontology updates can be time-consuming, automatic updates may not be convincing. With regard to COVID-19, vast knowledge has been learned from the literature and high throughput data analysis. Now the challenge becomes how to keep updating CIDO to have it remain up to date.

There have been different strategies proposed in terms of ontology updating. For example, the Principle of Minimal Change states that the knowledge lost during contraction should be minimal [4]. Solimando and Guerrini propose an ontology adaptation algorithm to fully automatically reformulate ontology axioms to adapt the condition when an entity in the ontology is deleted [5]. A framework of an iterative ontology update with minimal information loss using context-based reasoning method has been proposed [6]. These frameworks and methods emphasize the maintenance of existing ontology information and minimal context loss. To support ontology interoperability, the eXtensible Ontology development (XOD) strategy [7] emphasizes term reuse (instead of regenerating new terms, XOD1) and semantic alignment (XOD2), ontology design pattern usage for new term and axiom addition (XOD3), and community effort (XOD4).

In this paper, we applied the recursive usage of the XOD strategy for our CIDO updates by actively, progressively, and recursively identifying new knowledge from literature mining and manually annotated papers, or from our secondary data analysis of deposited data (ImmPort).

In our study, we extracted or performed secondary analysis on the ImmPort COVID-19 studies, applies the XOD strategy to the model and represent the experimentally verified results in the CIDO as a way to update and enhance CIDO. We also performed modeling and represent the learned knowledge in ontology. The whole process has been recursive because we do it periodically and consistently. Every time we do, we will improve the ontology through our recursive XOD-based ontology development and modeling.

Our study focused on the spike glycoprotein (S protein) and comparison in male and female responses to COVID-19. We include these two use cases because of their importance for coronavirus-host interactions and in disease outcome, respectively. The S protein of the SARS-CoV-2 plays a critical role in host-coronavirus interactions. The initial entry of the virus is driven predominantly by the S protein [8] which binds to the host cell’s ACE2 to initiate viral infection. This process is aided by transmembrane protease serine 2 (i.e., TMPRSS2) [9]. For this reason, S protein has been chosen as an antigen for several approved vaccines worldwide [10]. Additionally, males have consistently shown to have higher mortality and hospitalization rates in comparison to females [11]. We are using male and female as synonymous for biological sex unless otherwise specified as gender.

## Methods

### ImmPort secondary data analysis

The COVID-19 related papers were obtained from the ImmPort website (https://www.immport.org/shared/home). For each ImmPort COVID-19 study, ImmPort provides a list of structured information including a summary, design, adverse event, assessment, etc. Links to PubMed are also provided. All collected information was annotated, and a secondary data analysis was performed.

### Gene pathway analysis of sex comparison using ImmPort data using Reactome

The differentially expressed proteins in female or male COVID-19 patients compared to healthy controls were collected from Takahashi et al. [12] and mapped to a corresponding gene ID from a background list of their cytokine assay using binomial distribution. These genes cover different interferons, cytokines, chemokines, and growth factors. A significance cutoff of *p* < =0.05 was applied to the study results by secondary data analysis as shown in Extended Table 1 or Extended Table 2. The NCBI Gene names were then used in the Reactome pathway browser to identify the enriched pathways based on female or male gene lists.

**Table 1:** COVID-19 studies reported in ImmPort as of April 18, 2021

ImmPort Study ID	Title	PMID	Comments	Citation
SDY1667	Cross-reactive SARS-CoV-2 T cell epitopes in unexposed humans	32753554	Shared epitopes with common cold coronaviruses	[20]
SDY1640	T and B cell responses to SARS-CoV-2 coronavirus	32473127	Cytokine signatures/markers	[26]
SDY1662	An inflammatory cytokine signature predicts Covid-19 severity and survival	32839624	Inflammatory cytokine signatures	[30]
SDY1665	Longitudinal analyses reveal immunological misfiring in severe COVID-19 (Companion study to SDY1648)	32717743	Cytokine signatures	[31]
SDY1648	Sex differences in immune responses to SARS-CoV-2 (Companion study to SDY1655)	32846427	Overlapped genes with significant differences in female and male patients	[12]
SDY1641	Renin-angiotensin system inhibitors improve the clinical outcomes of COVID-19 patients with hypertension	32228222	RAS inhibitor drugs effective against hypertension	[49]

**Table 2:** Biomarkers of female and male COVID-19 patients and controls as reported in SDY1648

Gene ID	Gene Name	Significant Difference between Diseased & Healthy in the Same Sex^**a**^	Significant Difference in Female/male Patient^**a**^ After Adjustments	Significant Difference in Female/male in Health^**a**^ After Adjustments
1435	CSF1 colony stimulation factor 1	Both	- (Female)	- (Female)
1440	CSF3 colony stimulating factor 3	Female	- (Male)	- (Male)
3440	IFNa2 interferon alpha2	Both	- (Female)	- (Male)
3458	IFNG interferon gamma	Female	- (Female)	- (Male)
3557	IL-1RA interleukin 1 receptor antagonist	Both	- (Male)	- (Male)
3569	IL-6 Interleukin-6	Both	- (Male)	- (Female)
3606	IL-18 Interleukin-18	Female	- (Male)	- (Male)
3576	CXCL8 (IL8) C-X-C motif chemokine ligand 8	Female	- (Male)	- (Female)
3627	CXCL10 C-X-C motif chemokine ligand 10	Both	- (Male)	- (Female)
6351	CCL4 C-C motif chemokine ligand 4	Male	- (Male)	- (Female)
6352	CCL5 C-C motif chemokine ligand 5	Both	- (Male)	- (Female)

^a^ Statistically significant difference in female, male, or both female or male after adjusting for BMI and age. Terms in parenthesis indicates which was greater

### Recursive CIDO ontology updating of host-coronavirus interactions

**Fig. 1:**
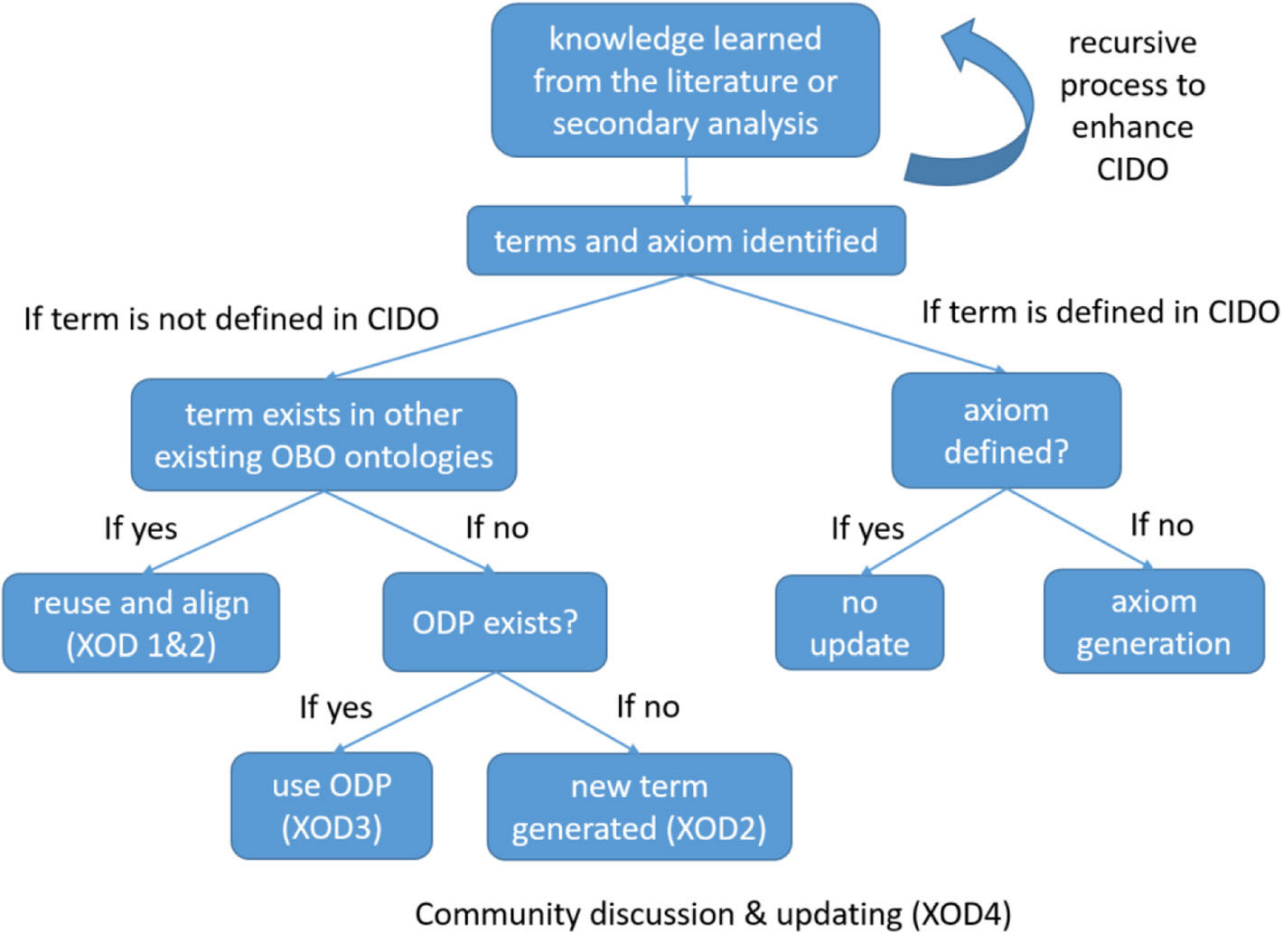
Strategy of CIDO updating using recursive XOD strategy. The recursive XOD strategy applies the use of the different principles of XOD design for use of new term generation along with creation of new axioms. Further knowledge either from literature or secondary analysis is used as a basis to generate new terms

Our CIDO ontology updating of host-coronavirus interactions followed a recursive eXtensible Ontology development (XOD) strategy [7] as laid out in Fig. 1. Specifically, after new knowledge is learned from the literature or secondary analysis, terms and axioms related to the knowledge are first identified. If the terms are not yet in the CIDO, we will: (i) identify, extract, and reuse the terms from existing ontologies (XOD1), and align them under CIDO (XOD2), (ii) generate new ontology terms using ontology design patterns (XOD3), and or (iii) manually add the terms to CIDO and align them to the semantic structure of existing CIDO (XOD2). Community-based discussion is also involved during the ontology development (XOD4). The XOD processes are done in a recursive way since new knowledge is iteratively and recursively added (Fig. 1).

Based on this strategy, CIDO reuses terms from existing ontologies and aligns all terms within a single semantic framework as defined by the Basic Formal Ontology (BFO) [13]. CIDO follows the Open Biological/Biomedical Ontologies (OBO) Foundry principles [14]. CIDO is an open source project with its source code available at https://github.com/CIDO-ontology/cido. CIDO is released under a Creative Commons 4.0 License. CIDO has been accepted as an OBO library ontology and has been deposited in the Ontobee ontology server [15], BioPortal [16], and OLS [17].

## Results

### ImmPort data exploration on the basis of existing CIDO development

The method implemented in our study is the recursive XOD strategy (Fig. 1). The added information should be semantically aligned with existing ontology structure. We applied recursive usage of the XOD development pipeline to continuously incorporate and integrate new knowledge data to CIDO.

**Fig. 2:**
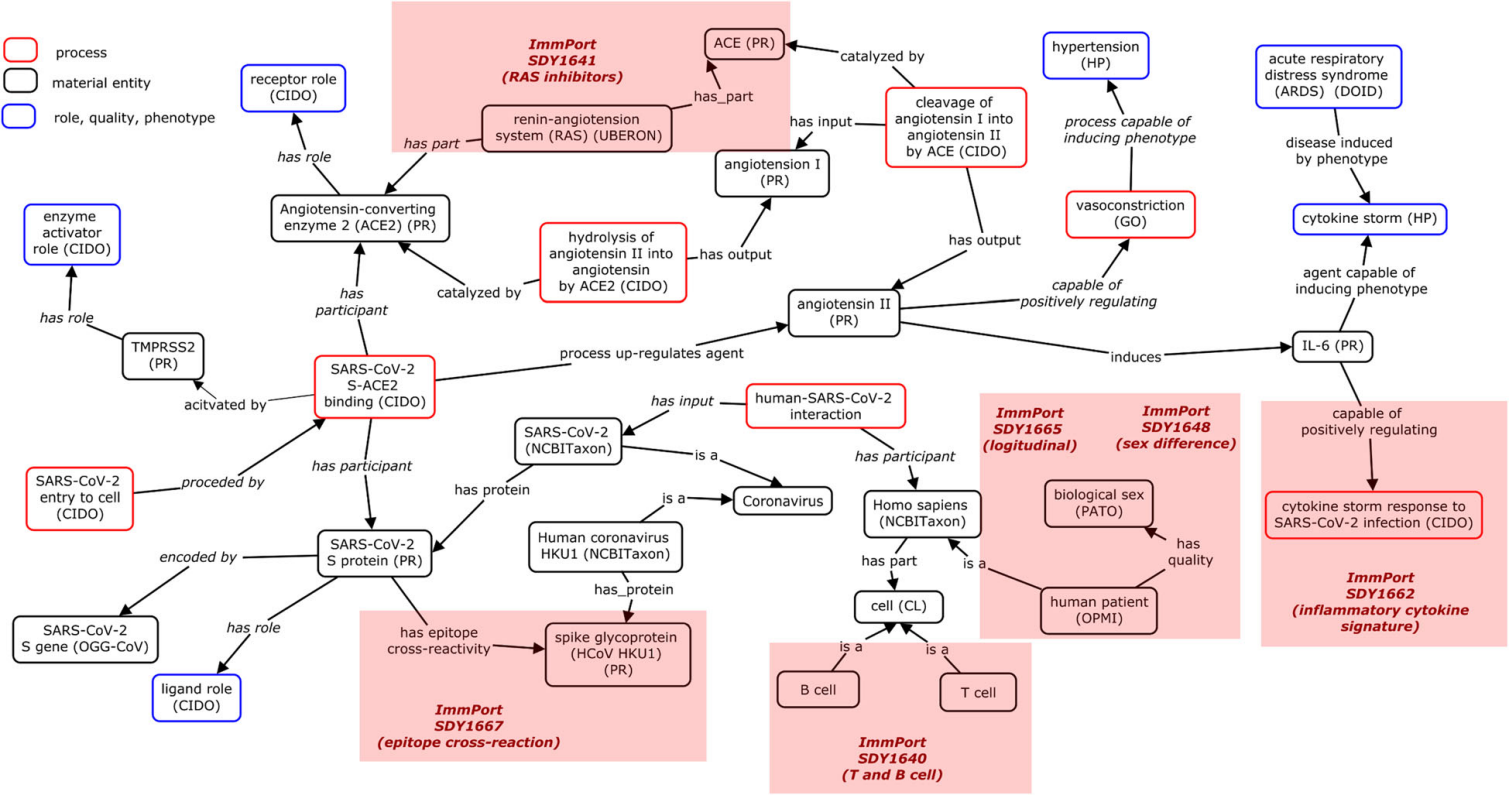
CIDO representation of host-coronavirus interactions and the addition of new knowledge learned from papers of ImmPort studies to CIDO. ImmPort studies are labeled with their study IDs (e.g., SDY1667). The termed highlighted are the terms newly added to incorporate the knowledge learned from the corresponding ImmPort studies. This figure shows how CIDO can be updated through our ontology reinforcement strategy

Figure 2 illustrates how CIDO has been developed and how ImmPort data can fit into the existing CIDO structure. A major task addressed in this study was to use the CIDO as the basis, add new knowledge learned from the ImmPort COVID-19 studies (Table 1) to the current version of CIDO, and then perform the secondary analysis to identify new scientific insights about host-SARS-CoV-2 interactions.

In our study, CIDO is used as a foundation and platform for semantic representation of host responses to COVID-19 infection. CIDO provides basic information about host-coronavirus interactions. Overall, SARS-CoV-2 viral processes include the viral binding to the host cell, entry to the cell, viral genetic replication, assembly of the virion, and release of the virion. CIDO also includes terms and axioms about immune responses to SARS-CoV-2 and has expanded modeling to account for dealing with unique changes from a pandemic. A portion of this information is represented in Fig. 2 showing parts of the SARS-CoV-2 life cycle including viral invasion to the host cell, viral replication, and viral shedding from the cell.

### CIDO representation of S protein focused coronavirus invasion, host response, and viral mutation

#### Modeling of viral invasion and host immune response by S protein

**Fig. 3:**
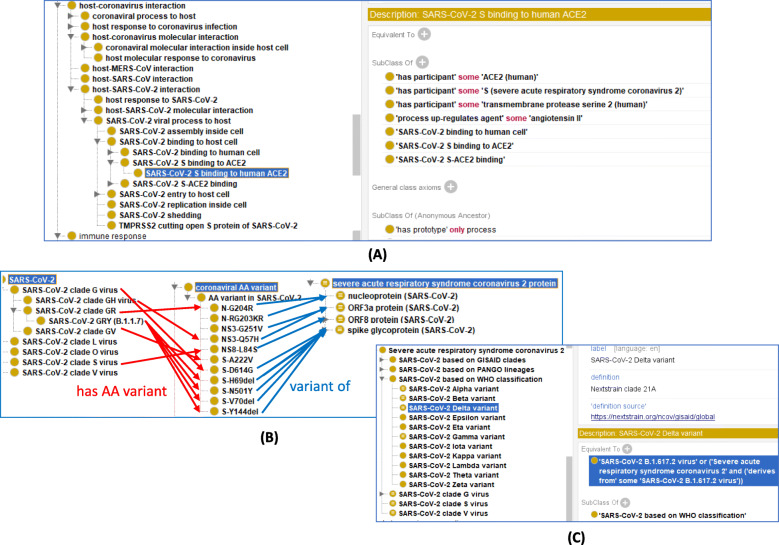
Ontological representation of S protein processes and variations. **a** CIDO ontological representation of viral life cycle in host (cell invasion, genetic replication, and release for cell and invading other cells) of virulent SARS-CoV-2. CIDO ontological representation of coronaviral molecular processes. The right bottom side of the screenshot represents different axioms for the SARS-CoV-2 S binding to human ACE2. **b** Seven SARS-CoV-2 clades are defined with each has its specific definition. For example, SARS-CoV-2 clade G virus has AA variant S-D614G, which is a variant of S protein of SARS-CoV-2. **c** CIDO representation of SARS-CoV-2 virus variants such as SARS-CoV-2 Delta variant based on the WHO classification

The S protein plays a key role in COVID-19 viral infection and disease pathology. Fig. 3a illustrates how the CIDO ontology represents various coronaviral processes on the surface of and inside the host, such as the main steps of viral infection, reproduction, and shedding (i.e., viral release out of host cell). The process of ‘SARS-CoV-2 S binding to human ACE2’ is defined with the following axioms:

*‘has participant’ some ‘ACE2 (human)’*

*‘has participant’ some ‘S protein of SARS-CoV-2)’*

*‘part of’ some ‘SARS-CoV-2 entry to human cell’*

*‘SARS-CoV-2 binding to human cell’*

*‘SARS-CoV-2 S-ACE2 binding’*

Such S-ACE2 binding is critical not only to the viral invasion, but also to the manifestation of many COVID-19 phenotypes such as pneumonia, hypertension, heart disease, acute kidney injury [18, 19].

Additionally, the issue of different clades brings about a concern of unique epitopes. A good representative study of their significance for ontology modeling is the study reported in ImmPort (SDY 1667) [20], which found 142 SARS-CoV-2 T-cell epitopes that are homologous epitopes to SARS-CoV-2 and multiple common cold human coronaviruses. Homologous epitopes are defined as any two epitopes, A and B, that exhibit sufficient homology and that when A elicits a host immune response and becomes part of the host immune memory, the A-specific memory will also recognize B. The Immune Epitope Database (IEDB) [21] has collected over 2000 known SARS-CoV-2-specific T or B cell epitopes, and the numbers are being updated every week. A consensus is that IEDB is the proper database to store and maintain the epitopes, and it is inappropriate for CIDO to record all the epitopes. Instead of listing all individual epitopes identified in the ImmPort study (SDY 1667) [20], we propose an ontology design pattern that represents the relation between two proteins that have epitope cross-reactivity. An example of such a representation is shown below:

*‘spike glycoprotein (SARS-CoV-2)’: ‘has epitope cross-reactivity’ some ‘spike glycoprotein (HCoV-OC43)’*

#### CIDO modeling of S protein mutation to avoid active host response

SARS-CoV-2 is a virus and by its nature undergoes under selective pressure that results in production of new variations within the viral proteins. For example, the B.1.1.7 clade variant that emerged in the UK [22]. B.1.1.7 already has early evidence for increased transmissibility and potential higher lethality [23]. While there are already ontological representations for immune responses to proteins in specific species, the actual representation of notable protein mutations has not previously been implemented. In Fig. 3a, we provide modeling on how this is done. Each mutant is identified by the protein name followed by a dash and the type of mutation. Identification of these proteins are done at the individual amino acid level as shown below.

*‘S-D614G’: ‘variant of’ some ‘spike glycoprotein (SARS-CoV-2)’*

S-D614G is interpreted as S protein with a missense mutation that causes the 614th amino acid, aspartic acid (D) to become glycine (G). CIDO has incorporated this to provide a standard set of annotations. A virus variant may have multiple mutations (Fig. 3b), which can also be systematically represented including an axiom as exemplified below:

*‘SARS-CoV-2 GRY (B.1.1.7): ‘SARS-CoV-2 clade GR virus’ and (‘has AA variant’ some (S-H69del and S-V70del and S-Y144del and S-N501Y and N-G204R))*

One of the challenges for CIDO has been the ontological classification of new SARS-CoV-2 lineages and strains that have emerged. Multiple naming schemas exist, each with different criteria for their categories. The World Health Organization (WHO) has designated certain coronaviruses as either variants of concern or variants of interest and named them using as Alpha, Beta, Gamma, Delta, etc. (Fig. 3c). For example, the SARS-CoV-2 Delta variant is defined as an equivalent axiom:

*SARS-CoV-2 Delta variant: ‘SARS-CoV-2 B.1.617.2 virus’ or ('Severe acute respiratory syndrome coronavirus 2’ and (‘derives from’ some ‘SARS-CoV-2 B.1.617.2 virus’))*

Here *‘SARS-CoV-2 B.1.617.2 virus’* is a variant classification based on Phylogenetic Assignment of Named Global Outbreak Lineages (PANGO) [24]. The relation ‘derives from’ indicates that the Delta variant includes the SARS-CoV-2 B.1.617.2 virus or any other viral variant derived from the virus. In addition, CIDO also represents the variant classification assigned by the organization of Global Initiative on Sharing Avian Influenza Data (GISAID) [25] (Fig. 3c).

### CIDO representation of RAS related drug interruption for treating COVID-19

The ImmPort study (SDY1641) investigated the roles of renin-angiotensin system (RAS) inhibitors, including angiotensin-converting enzyme inhibitors and angiotensin II receptor blockers, in treating COVID-19 patients with hypertension [26]. Patients treated with angiotensin-converting enzyme (ACE) inhibitors or angiotensin II receptor blocker had a lower rate of severe diseases and lowered IL-6 in peripheral blood. The ACE inhibitors or angiotensin II receptor blocker therapy also increased the CD3 and CD8 T cell counts in peripheral blood and decreased the peak of viral load compared to other antihypertensive drugs [26].

RAS is also closely associated with coronavirus S protein since the S protein binds to the host angiotensin-converting-enzyme 2 (ACE2), a key RAS component. The binding between the S glycoprotein and ACE2 needs to be activated by TMPRSS2, a cellular receptor [9] (Fig. 2). Such binding leads to the subsequent downregulation of ACE2 [27, 28]. angiotensin-converting enzyme inhibitors inhibit the activity of ACE, an important component of the RAS that converts angiotensin I to angiotensin II. Therefore, angiotensin-converting enzyme inhibitors decrease the formation of angiotensin II, a vasoconstrictor. Angiotensin II receptor blockers bind to and inhibit the angiotensin II receptor type 1 (AT1), a receptor that has vasoconstriction role. Angiotensin II receptor blockers can then block the activation of the AT1 and prevent the binding of angiotensin II, leading to the treatment of hypertension [29].

**Fig. 4:**
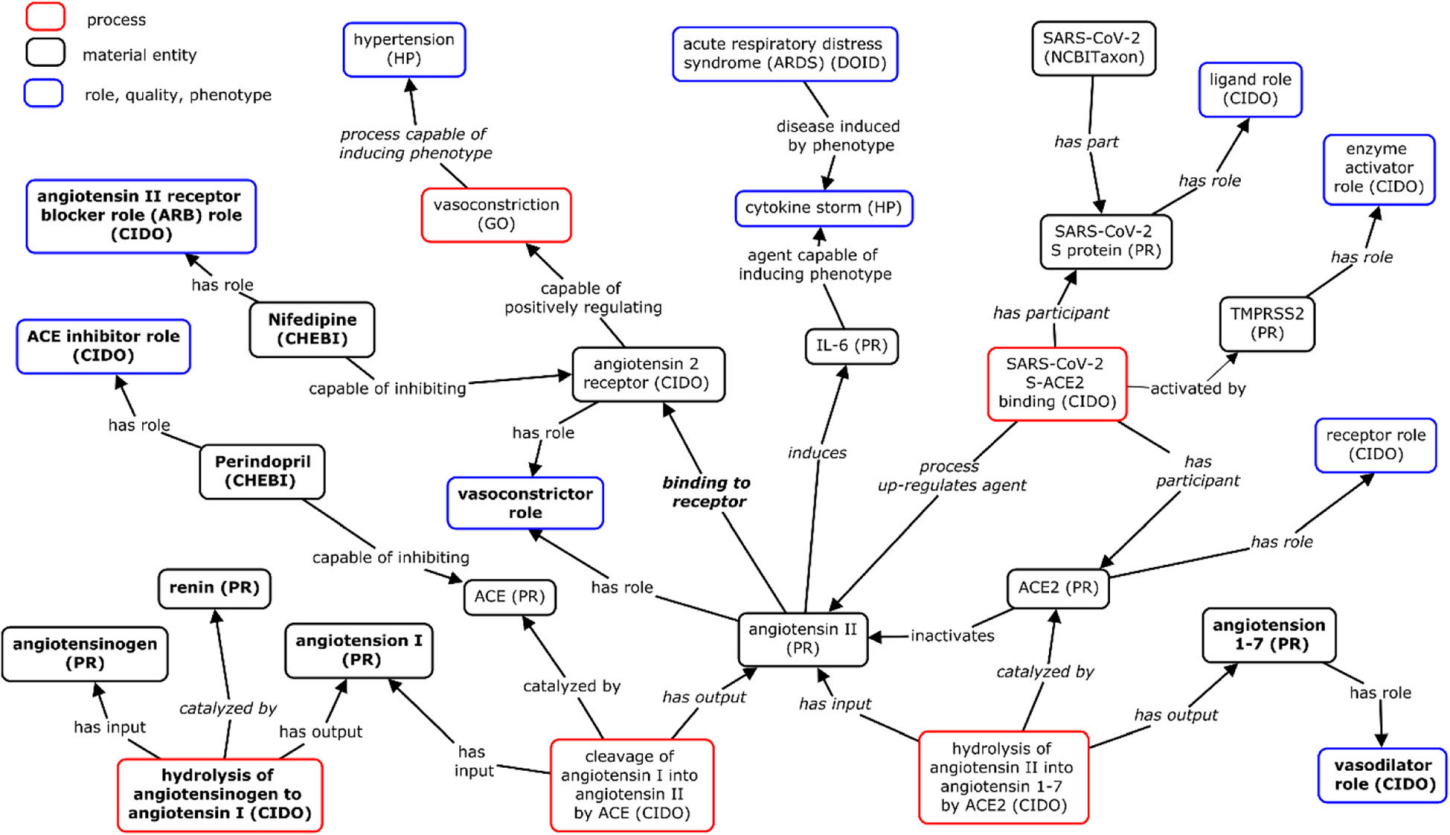
Ontological representation of RAS pathway and drug roles. The bold text represents newly added terms from the ImmPort-focused data annotations

We have modeled the above RAS-related process as shown in Fig. 4. Note that many terms and axioms were already represented in our previous CIDO modeling. To add new results obtained from the ImmPort study (SDY1641), multiple new terms and axioms were added as seen in the bold terms in Fig. 4. In our CIDO modeling, we defined many roles, such as ‘ACE Inhibitor role’, ‘angiotensin II receptor blocker role’, ‘vasoconstrictor role’, and ‘vasodilator role’. These roles can be then used to annotate different drugs or molecules, for example:

*perindopril: ‘has role’ some ‘ACE inhibitor role’*

*nifedipine: ‘has role’ some ‘angiotensin II receptor blocker role’*

By doing so, the biological relevance of the drugs and molecules can be clearly noted and understood by humans and computers.

### CIDO representation of host immune markers between immune profiles and covariates that correlate with COVID-19 outcomes

There are many host immune markers that correlate with COVID-19 outcomes. Figure 4 shows the general pattern of gene expression patterns in an ontological representation of genes (including gene markers) that are susceptible to be up-regulated under a specific condition such as SARS-CoV-2 infection.

Two ImmPort publications from two studies report host immune markers that correlate with COVID-19 outcomes: one that introduce inflammatory cytokine signatures that predicts COVID-19 severity and survival (ImmPort Study SDY1662) [30], and the other that introduces many more immune signatures associated with severe COVID-19 (ImmPort Study SDY1665) [31]. The first paper [30] demonstrates that IL-6 and TNF-alpha both are strong independent predictors of disease severity and death outcomes, with IL-18 also serving as a strong, but not independent predictor. Higher levels of IL-6 elevation are associated with the cytokine release syndrome (CRS), a condition that the SARS-CoV-2 infection also causes in compared to higher immune control [32]. The second paper [31] generated an immune profile by analyzing the immune responses in 113 patients with moderate or severe COVID-19, uncovering an overall increase in innate cell types and a concomitant reduction in T cell number. Severe COVID-19 was found to be associated with the elevation of cytokines and immune pathways associated with type 1 (antiviral), type 2 (anti-helminths), and type 3 (antifungal) type II pathways and higher levels of growth factors, type 1/2/3 cytokines and chemokines. However, patients with moderate COVID-19 had a progressive reduction in type 1 (antiviral) and type 3 (antifungal) responses after an early increase in cytokines and enriched with growth factors [31].

The initial immune signature of IL-6 for COVID-19 disease pathology have been further investigated and associated with other pathologies. For example, COVID-19 is linked to cytokine release syndrome (CRS), and the pathogenesis of CRS is associated with IL-6-mediated production of hyperinflammatory cytokines and plasminogen activator inhibitor-1 (PAI-1) [32]. The inhibition of IL-6 signaling using tocilizumab decreased PAI-1 production and alleviated the clinical symptoms in severe COVID-19 patients [32]. However, Kang et al. [32] also shows that while still elevated compared to healthy control, IL-6, IL-8, and MCP-1 are lower to other CRS diseases. Children had three cytokines increased interferon (IFN)-γ-induced protein 10 (IP10), interleukin (IL)-10 and IL-16 [33].

To model these results, we implemented a new class for biomarker and immune signature. A biomarker is a material entity that has a change in expression associated with a specific response to some specific biological process. An immune signature is a biomarker for some specific disease process. We included new object relations to model these differences for different SARS-CoV-2 disease processes.

*IL-6: ‘up-expressed as immune signature of’ some (‘severe COVID-19 disease’ and ‘death stage’)*

**Fig. 5:**
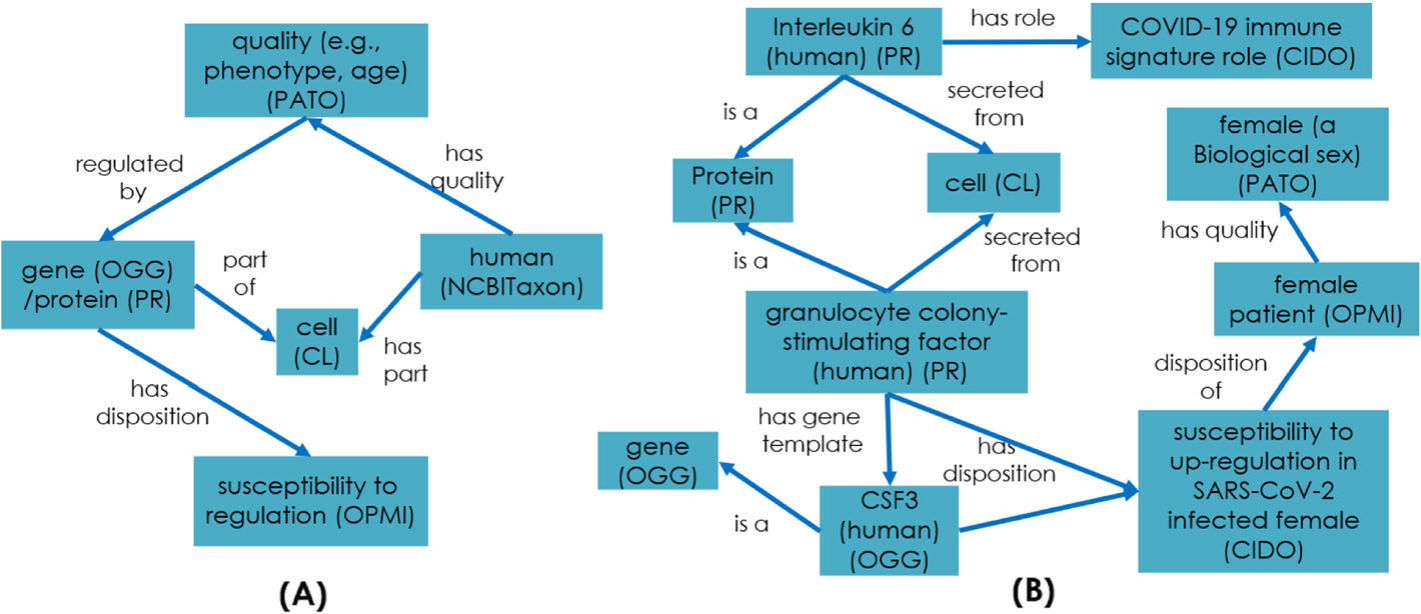
Ontological representation of gene signature and quality-based immune responses. **a** General pattern of gene expression patterns in human. **b** CIDO representation of gene expression patterns in SARS-CoV-2 infected patients

However, these immune markers and profiles are also dependent on host qualities. Figure 5a shows that different qualities, such as biological sex (F/M), age, comorbidities, will infect disease outcomes. Figure 5b provides an CIDO representation of gene expression patterns in SARS-CoV-2 infected patients. Here we focus on the sex comparisons an example to illustrate the effect of biological sex to the disease outcome.

Increasing evidence show that male sex is a risk factor for a more severe COVID-19 disease outcome [12]. In one of the early studies with data in Wuhan, China, of 86 male COVID-19 patients, 12.8% (11/86) died; in comparison, of 82 female patients, 7.3% (6/82) died [34]. A cohort study of 17 million COVID-19 adult patients in England reported a strong association between male sex and risk of death [35]. Globally, approximately 60% of COVID-19 associated deaths are reported in men [36].

In CIDO, we represent the high susceptibility of male to the death using the following axiom:

*‘male infected with SARS-CoV-2’: ‘has increased susceptibility compared to female to’ some ‘death stage’*

**Fig. 6:**
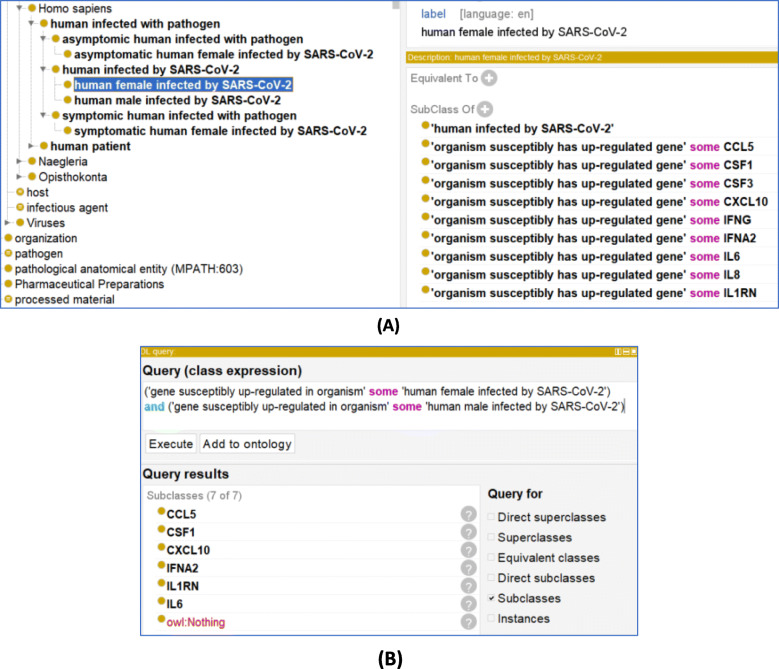
Sex differences in gene ontology and DL query. **a** CIDO ontological representation of sex-based immune response for SARS-CoV-2. The genes listed are chosen from the results in Table 2. **b** DL Query infers properties provide a list to identify shared genes between males and females

This raises important question on the underlying molecular mechanisms underlying this sex difference and prompted further investigation using secondary analysis from the ImmPort studies. A total of 11 genes from Takashi et al. [12] were collected and compared for age and Body Mass Index corrected differences between patients and health care workers for each sex and is shown in Table 2. From this gene list, males and females showed statistically significant increases in 7 and 10 genes, respectively. To represent these differences between individuals (sex, exposure), we added new CIDO terms to distinguish between these differences as illustrated below (Fig. 6a).

*‘symptomatic human male infected by SARS-CoV-2’: ‘organism susceptibly has up-regulated gene’ some ‘CCL4’*

Such modeling allows us to perform semantic query as exemplified in Fig. 6c. In this example, we used a DL query to easily identify the number of up-regulated genes that are shared by male and female patients with COVID-19 (Fig. 6b).

In addition to the gene list identification and modeling, we further performed a secondary data analysis on the pathways. These gene lists were placed into Reactome to generate a set of pathways they were enriched. From these pathways, we restricted the background to 58 cytokines mapped from Takashi et al. [12] (out of 61 assays) and found that IL-10 immune pathways in both males and females were shown to be significant (*p* value of 8.47E-5 and 3.56E-5, respectively) despite differences in genes. The implication of such result is described in the following Discussion section.

## Discussion

We have provided multiple contributions to the community with this effort. First, we demonstrated how we can apply a recursive XOD strategy to improve the CIDO by incorporating new knowledge learned from 6 publications from 6 ImmPort Studies related to COVID-19. Secondly, we demonstrate our better understanding of host responses to COVID-19, including sex differences, inflammatory cytokine responses, etc. Third, we performed secondary data analysis on the sex differences in response to COVID-19 infection. Our work showed that pathway analysis could provide more information than the gene list studies. Lastly, we provide a DL query to demonstrate how the integrated work can provide reasoning and inferences. We showed that our ontology framework supports knowledge representation, data and metadata standardization, and information query and analysis.

Our study presents a feasible strategy for new ontology updates by actively and progressively identifying new knowledge from literature mining and manual annotation of papers, or from our secondary data analysis of deposited data such as ImmPort. This is a process we need to do recursively. With each recursion, we reinforce the ontology coverage and quality. The basic XOD strategy informs how the CIDO can be developed, but it does not specifically inform how CIDO can be updated. For COVID-19 studies, expansive knowledge has been introduced by the literature and databases in a short period of time. Therefore, representing such data using CIDO has become challenging for our CIDO development team. Here we practice the XOD strategy and show that recursive XOD processes can help update the ontology. Instead of adopting a minimal updating principle for rapidly changing data, we used an active update pipeline. The information from other databases may also be incorporated.

New scientific insights about COVID-19 were also identified from the literature, summarized, and integrated into CIDO, leading to possible reasoning. For example, we were able to merge the epitope knowledge with the virus hierarchy, and virus variant information. It has been found that that many humans can develop protection against COVID-19 infection even when they have not been exposed to the SARS-CoV-2 virus, and such a phenomenon was owing to their protection to exposure to a common cold coronavirus and the cross reactivity of the epitopes between SARS-CoV-2 and some common cold coronaviruses [20, 26]. The viral variants also demonstrate the differences in COVID-19 transmissibility, vaccine efficacy and treatment efficacy [37, 38]. These findings have been semantically recorded in our CIDO knowledge representation.

Biomarkers and qualities (such as sex) in COVID-19 have been shown to manifest in negative disease outcomes (hospitalization, ICU, and mortality) and in differences in immune responses [11]. For example, age as an influence on comorbidities as a covariate for fatality is strongly sex specific [34]. Our secondary analysis using Reactome was inconclusive in most immune pathways; however, our Reactome study showed that both male and female patient populations had enriched expression of the IL-10 signaling pathway. The enrichment of the IL-10 signaling pathway is a novel finding from our secondary analysis of the ImmPort data since it was not reported in the original paper [12]. This prompted the next step of the recursive XOD to incorporate this new knowledge by looking to incorporate new terms and axioms related to IL-10. A recent March paper has proposed a hypothesis that IL-10 up-regulation is responsible for COVID-19 uniqueness in comparison to other coronaviruses [39]. SARS differs from COVID-19 in that IL-10 is only increased in convalescent SARS-CoV patients and but not with other SARS disease phenotypes [40, 41]. The IL-10 signaling pathway has many effects, and through direct macrophage and monocyte cells the pathway affects T-cell development and differentiation while enhancing B cell immune response [42]. Such mechanism appears to exist in both genders of patients, suggesting that it is a sex-independent mechanism. The authors also acknowledge that our finding is limited by the short list for background enrichment; however, multiple other papers have consistently found differences in outcomes [43, 44].

Despite strong evidence showing differences between sex, many countries have disaggregated COVID-19 data to sex which would occlude relations caused by differences in sex [45]. The modeling of different dispositions occurring due to differences in sex is able to help specify these tends which are not otherwise modeled in other ontologies.

The emergence of these new strains, such as B.1.1.7 and B.1.167+ [46] represent an important driver to our continuous application of the recursive XOD strategy for the future CIDO development. The modeling of characteristics of disease severity from their biomarkers and demographic differences are important additions to CIDO. Further incorporation of the transcriptome can be used to identify further molecular mechanisms and help elucidate further interactions in SARS-CoV-2.

As a formal ontology, CIDO is a logic computer-interpretable way of coronavirus-related knowledge representation, which supports reasoning and inference. CIDO does not represent specific instance-level data such as the data in the ImmPort database. Instead, CIDO represents coronavirus-related knowledge learned from the peer-reviewed publications or collected in well-annotated databases. On the other hand, ontology can support database data representation by ontologically representing the metadata or specific knowledge. For example, our group also maintains and develops the Vaccine Ontology (VO) [47, 48], which has been adopted and used in the ImmPort vaccine data annotation. This project is currently funded by an ImmPort-related NIH grant. Our ontology modeling outcomes are being shared with ImmPort, and we look forward to collaborating with the ImmPort team on how possibly our new CIDO knowledge representation can support their data storage and modeling.

## Conclusion

ImmPort COVID-19 studies were further analyzed, and the knowledge learned were modeled and represent in the CIDO ontology. A recursive XOD strategy was proposed to systematically add the new knowledge to CIDO. New use cases include COVID-19 strain representation, the epitope cross-reactivity information and showing sex comparisons in responses to COVID-19 infection. Our use case studies demonstrate how we can actively and recursively update CIDO without suffering logical misinformation. Based on existing CIDO representation of various coronaviruses and proteins, we were able to quickly add the new knowledge of shared immune epitopes in different proteins of SARS-CoV-2 and other human coronaviruses that cause common colds.

## Data Availability

All data generated or analyzed during this study are included in this published article and available as part of ImmPort or at https://github.com/CIDO-ontology/cido.

## Abbreviations

BFO: Basic Formal Ontology
ChEBI: Chemical Entities of Biological Interest
CIDO: Coronavirus Infectious Disease Ontology
DL query: Description Logics query
DOID: Disease Ontology
DRON: Drug Ontology
GISAID: Global Initiative on Sharing Avian Influenza Data
GO: Gene Ontology
IAO: Information Artifact Ontology
NCBITaxon: NCBI organismal classification
OBCS: Ontology of Biological and Clinical Statistics
OBI: Ontology for Biomedical Investigations
OBO: The Open Biological and Biomedical Ontologies
OWL: Web Ontology Language
PANGO: Phylogenetic Assignment of Named Global Outbreak Lineages
PATO: Phenotypic Quality Ontology
PR: Protein Ontology
RDF: Resource Description Framework
SPARQL: SPARQL Protocol and RDF Query Language
UBERON: Uberon multi-species anatomy ontology
